# Influence of a ward-based pharmacist on the medication quality of geriatric inpatients: a before–after study

**DOI:** 10.1007/s11096-021-01369-1

**Published:** 2022-01-25

**Authors:** Esther Katharina Kiesel, Michael Drey, Yvonne Marina Pudritz

**Affiliations:** 1 Technical University of Munich, Hospital Pharmacy, University Hospital rechts der Isar, Munich, Germany; 2 University Hospital, Doctoral Programme Clinical Pharmacy, LMU Munich, Munich, Germany; 3 University Hospital, Department of Medicine IV, LMU Munich, Munich, Germany; 4 University Hospital, Hospital Pharmacy, LMU Munich, Munich, Germany

**Keywords:** Aged, Clinical pharmacist, Geriatrics, Hospital, Medication reconciliation, Pharmacy service

## Abstract

*Background* Despite several international studies demonstrating that ward-based pharmacists improve medication quality, ward pharmacists are not generally established in German hospitals. *Aim* We assessed the effect of a ward-based clinical pharmacist on the medication quality of geriatric inpatients in a German university hospital. *Method* The before-after study with a historic control group was conducted on the geriatric ward. During the control phase, patients received standard care without the involvement of a pharmacist. The intervention consisted of a clinical pharmacist providing pharmaceutical care from admission to discharge. Medication quality was measured on admission and discharge using the Medication Appropriateness Index (MAI). A linear regression analysis was conducted to calculate the influence of the intervention on the MAI. *Results* Patients in the intervention group (*n* = 152, mean 83 years) were older and took more drugs at admission compared to the control group (*n* = 159, 81 years). For both groups, the MAI per patient improved significantly from admission to discharge. Although the intervention did not influence the summated MAI score per patient, the intervention significantly reduced the MAI criteria *Dosage* (*p* = 0.006), *Correct Directions* (*p* = 0.016) and *Practical Directions* (*p* = 0.004) as well as the proportion of overall inappropriate MAI ratings (at least 1 of 9 criteria inappropriate) (*p* = 0.015). *Conclusion* Although medication quality was already high in the control group, a ward-based clinical pharmacist could contribute meaningfully to the medication quality on an acute geriatric ward.

## Impacts on Practice

- Ideally, ward-based clinical pharmacists should be fully integrated members in the multi- professional team taking care of geriatric patients to ensure continuous pharmaceutical care.
- Patients profit from the additional provided pharmaceutical care through reduced drug-related problems.
- Further research should look at the economic impact such as reduction of workload for medical and nursing staff and reduced admission cost to provide an argument for establishing further ward-based clinical pharmacist posts in German secondary care.

## Introduction

Multimorbidity of geriatric patients often leads to polypharmacy and to a higher probability of interactions, side effects and other drug-related problems (DRP) [1]. Geriatric patients are more susceptible to adverse drug reactions (ADR) [2, 3]. Polypharmacy and DRP are often causes of hospital readmissions of elderly patients [4–6]. In geriatric care, the physicians focus on polypharmacy and improving medication [7, 8]. Nevertheless, the addition of pharmaceutical expertise improved medication quality even further [9].

Although it is known that pharmaceutical care delivered by clinical pharmacists with a secondary care setting can reduce drug related problems (DRP), potentially inappropriate medication (PIM) and in subgroups even readmission rates, there is only very little data available on hospital pharmacists’ effect in geriatric care in Germany [10–12]. All studies found were conducted without a control group [13, 14] or restricted to retrospective data analysis without pharmaceutical interventions [15, 16]. A recently published study in Germany demonstrated that a pharmacist’s intervention successfully reduced DRP in geriatric inpatients [17]. A Europe-wide systematic literature review resulted in several studies showing a positive effect of pharmacists on medication quality and safety of older inpatients [18]. More complex interventions like pharmaceutical care from admission to discharge were favourable, as this kind of intervention can both reduce medication errors at admission through medication history, improve the appropriateness of prescribing and might reduce drug-related readmissions through discharge management [18]. Not only in geriatric care, but also in general, clinical pharmacy services in Germany are still developing and there is a gap in services when compared with other countries. Especially ward-based clinical pharmacists are not routinely utilised in secondary care [19].

### Aim

In this study, we assessed the effect of a ward-based clinical pharmacist on the medication quality of geriatric inpatients in a German university hospital.

### Ethics approval

The ethics committee of the institution approved the study protocol [No. 20–1053, final version 19th January 2021].

## Method

The study was conducted on the geriatric ward of a university teaching hospital in Germany (LMU, Munich). The unit comprises 20 beds for patients aged 65 and older who present with acute geriatric problems and rehabilitation potential. It was established December 2014. A multi-professional team composed of a geriatrician, residents, nurses, physiotherapists, occupational therapists, speech therapist, a social worker, and a psychologist is present on the ward. The study was a before-after study with a historic control group. During the control phase, no pharmacist was involved in the medical care and patients received standard care.

The intervention consisted of a clinical pharmacist (EKK) providing pharmaceutical care from admission to discharge. The pharmacist was present on the ward on weekdays from 8 am to 4 pm and had access to patient medical records as well as direct contact with patients and caregivers. For every patient, the pharmacist performed a medication reconciliation at admission and created a medication plan at discharge that was given to the patient together with the doctor’s discharge letter. In addition, the pharmacist performed a daily review of all prescriptions and joined medical rounds once a week. The pharmacist was available for ward staff to help with any questions during the intervention period.

All patients admitted to the unit between August 2015 and January 2016 were retrospectively evaluated for eligibility for the control group. Exclusion criteria were patients younger than 65 years or not discharged to the primary care setting from the ward (*e.g.* due to transfer to another ward or hospital or death or discharged against medical advice).

All patients admitted to the unit between May 2018 and December 2018 were retrospectively evaluated for eligibility for the intervention group. Additional exclusion criteria for the intervention group were: pharmacist couldn’t perform medication reconciliation at admission and absence of the pharmacist during the stay on ward.

The clinical pharmacist performed a medical record review to determine demographic characteristics and medications. Results from geriatric assessment, like activities of daily living (ADL), were documented [20]. The Charlson comorbidity score (CCS) was calculated [21]. Data were collected for both groups.

Appropriateness of prescribing was measured on admission and discharge. The Medication Appropriateness Index (MAI) was selected because it is currently the most comprehensive instrument for evaluating appropriateness [22]. It is a common tool for measuring medication quality and has been used in similar studies in Europe [18]. The MAI consists of 10 criteria, of which nine were used. The ratings generate weighted scores that serve as summary measures of prescribing appropriateness (0–17 per drug; the higher the score, the more inappropriate the rating). The total MAI score per patient can be obtained by summing up the MAI scores of all drugs prescribed for an individual patient. The criterion *Cost* was not used as medication costs differ widely between primary and secondary care in Germany. This approach for using the MAI is common in German settings [17].

The main investigator (EKK) evaluated the prescribing of all regularly scheduled medications according to the MAI. For both groups, a second investigator (YMP) evaluated the MAI independently for a random sample (*n* = 29) of patients to validate the assessment. Interrater reliability was tested with Kappa.

Additional outcome measures were collected after discharge: the proportion of drugs listed as PIM as labelled by Beers [23], Priscus [24] or FORTA (Fit fOR The Aged) [25]. For Beers criteria all unconditional PIM were used (Table 3 in Beers publication) [23].

All identified DRP and the resulting interventions during the intervention period were documented using the classification of the Pharmaceutical Care Network Europe (PCNE V8.01) [26] and categorized according to the National Council for Medication Error Reporting and Prevention (NCCMERP) (Table 1) regarding the severity of its consequences [27]. The main investigator did all PCNE and NCCMERP classifications and a second investigator validated a random sample (*n* = 72). Both investigators assessed independently all unclear classifications and all NCCMERP classifications resulting in harm to the patient (E–I).

**Table 1:** NCCMERP categories [31]

Category	Definition
A	Circumstances or events that have the capacity to cause error
B	An error occurred but the error did not reach the patient
C	An error occurred that reached the patient but did not cause patient harm
D	An error occurred that reached the patient and required monitoring to confirm that it resulted in no harm to the patient and/or required intervention to preclude harm
E	An error occurred that may have contributed to or resulted in temporary harm to the patient and required intervention
F	An error occurred that may have contributed to or resulted in temporary harm to the patient and required initial or prolonged hospitalization
G	An error occurred to or resulted in permanent patient harm
H	An error occurred that required intervention necessary to sustain life
I	An error occurred that may have contributed to or resulted in the patient’s death

A sample size estimation was calculated: 140 patients per group was deemed necessary to detect an improvement in MAI difference from admission to discharge of 4 points with a power of 80% and a significance level of 0.05. To adjust for missing data in the retrospective evaluation, a group size of 250 patients was determined. As the study ward treats around 250 patients in 6 months, a study period of 6 months was specified. To adjust for planned absences of the study pharmacist (*e.g.* annual leave), the intervention period was set at 8 months.

Study groups at baseline were compared using chi-square for categorical variables and the Mann–Whitney-U test for continuous variables. Baseline and discharge ratings were compared within groups, using Wilcoxon test. A linear regression analysis was conducted to calculate the influence of the intervention on MAI, independent of other variables like age, sex, CCS and number of drugs at admission. In each test, statistical significance was considered to be 0.05. Statistical analyses were performed using SPSS Statistical Software 25.0.0.1 (SPSS Inc., Chicago, IL).

## Results

**Fig. 1:**
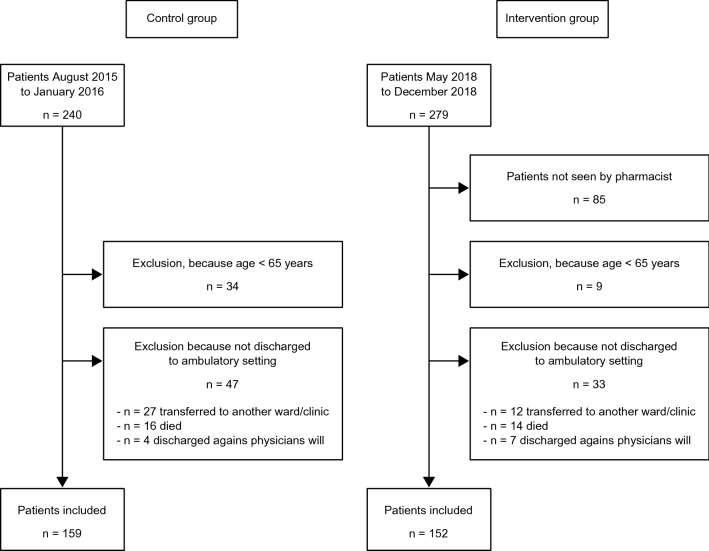
Flow of patients in control and intervention group

Due to age and not discharged to the primary care setting, 159 patients were included in the control and 152 in the intervention group. Figure 1 summarizes the flow of patients admitted to the ward and inclusion in the study. The patients of the intervention group were older and took more medications at admission (Table 2). The sex distribution, CCS, ADL, and MAI at admission were comparable between the two groups.

**Table 2:** Patient characteristics in control and intervention group

	Control group (n = 159)		Intervention group (*n* = 152)		*P*-value	
*Demographics*						
Age	81 ± 7		83 ± 7		**0.021**	
Female, n (%)	96 (60)		97 (64)		0.532	
*Clinical status and pharmaceutical data at admission*						
Charlson comorbidity score	2.0 ± 1.6		2.4 ± 1.9		0.057	
ADL (Barthel Index)	47 ± 22 (*n* = 98)		42 ± 16 (*n* = 113)		0.138	
Drugs	7 ± 4		8 ± 4		**0.004**	
MAI summated patient score	18 ± 14		20 ± 14		0.168	
	Admission	Discharge	*P*-Value	Admission	Discharge	*P*-Value
MAI per drug	2.6 ± 1.7	1.5 ± 1.0	** < 0.001**	2.4 ± 1.5	1.5 ± 1.0	** < 0.001**
MAI summated patient score	18 ± 14	12 ± 10	** < 0.001**	20 ± 14	14 ± 10	** < 0.001**
	Admission (*n* = 1038)	Discharge (*n* = 1197)	*P*-Value	Admission (*n* = 1254)	Discharge (*n* = 1438)	*P*-Value
*MAI criteria (%)*						
Indication	17.7	7.8	** < 0.001**	17.5	10.0	** < 0.001**
Effectiveness	23.8	12.1	** < 0.001**	21.8	12.9	** < 0.001**
Dosage	15.3	12.4	0.050	13.0	8.1	** < 0.001**
Directions correct	9.2	10.3	0.415	9.4	7.0	**0.020**
Directions practical	27.9	27.0	0.614	27.7	21.8	** < 0.001**
Drug-Drug Interactions	3.4	2.5	0.225	2.2	1.9	0.611
Drug-Disease Interactions	12.1	6.9	** < 0.001**	11.8	7.0	** < 0.001**
Duplication	1.1	0.3	**0.036**	1.1	0.6	0.168
Duration	18.9	10.1	** < 0.001**	18.8	12.4	** < 0.001**
Overall*	60.4	49.2	** < 0.001**	54.4	42.6	** < 0.001**
*Potentially inappropriate medicines (%)*						
Beers	7.7	4.6	**0.002**	6.1	3.5	**0.002**
PRISCUS	1.7	1.1	0.191	2.0	1.0	**0.027**
FORTA-D	1.6	0.6	**0.016**	1.9	0.5	**0.001**

Unless indicated otherwise, reported numbers are mean ± standard deviation. All *P*-values < 0.05 are considered significant and printed bold. Number of drugs includes all regular/scheduled drugs on admission (prescribed and non-prescribed). MAI at admission and at discharge and percentage of drugs with inappropriate ratings on admission and at discharge using the MAI and percentage of drugs listed on lists with potentially inappropriate medicines. * = inappropriate rating in at least 1 of the 9 criteria, ADL = activities of daily living

MAI improved significantly in both groups from admission to discharge, both calculated as MAI per drug and as MAI summated patient score (Table 2). In the control group, the MAI criteria *Indication, Effectiveness, Drug-Disease Interactions, Duplication* and *Duration* as well as the proportion of overall inappropriate MAI ratings (at least 1 of 9 criteria inappropriate) improved significantly from admission to discharge (Table 2). In addition, the criteria *Dosage, Correct Directions,* and *Practical Directions* improved significantly in the intervention group from admission to discharge. While the proportion of medications listed in the Beers list and medications rated FORTA-D decreased significantly both in the control and in the intervention group, the proportion of medications listed in PRISCUS list decreased significantly only in the intervention group (Table 2). A total of 29 patients were doubly assessed with a Kappa of 0,59, any discrepancies were resolved by discussing cases individually.

Linear regression analysis shows in all models a significant effect for the intervention for the MAI criteria *Dosage, Correct Directions,* and *Practical Directions* as well as for the proportion of overall inappropriate MAI ratings (at least 1 of 9 criteria inappropriate) (Table 3). No significant effect of the intervention on the proportion of medications on the Beers list, PRISCUS list and FORTA-D could be demonstrated (Table 3).

**Table 3:** Results from linear regression analysis for intervention effect

	Model 1	Model 2	Model 3	Model 4
MAI summated patient score	**0.115 (0.043)**	0.063 (0.215)	0.065 (0.199)	0.056 (0.276)
*MAI criterion*				
Indication	**0.131 (0.022)**	0.091 (0.096)	0.099 (0.071)	0.080 (0.146)
Effectiveness	0.061 (0.285)	0.050 (0.359)	0.050 (0.354)	0.034 (0.540)
Dosage	**− 0.158 (0.006)**	**− 0.166 (0.004)**	**− 0.161 (0.005)**	**− 0.160 (0.006)**
Directions correct	**− 0.138 (0.016)**	**− 0.141 (0.017)**	**− 0.143 (0.016)**	**− 0.134 (0.026)**
Directions practical	**− 0.162 (0.004)**	**− 0.185 (0.002)**	**− 0.178 (0.003)**	**− 0.173 (0.004)**
Drug-Drug Interactions	**− **0.034 (0.558)	**− **0.050 (0.403)	**− **0.064 (0.282)	**− **0.068 (0.151)
Drug-Disease Interactions	0.048 (0.400)	0.041 (0.446)	0.033 (0.538)	0.048 (0.378)
Duplication	0.060 (0.292)	0.066 (0.232)	0.068 (0.218)	0.072 (0.204)
Duration	**0.118 (0.039)**	0.082 (0.140)	0.092 (0.097)	0.080 (0.153)
Overall*	**− 0.138 (0.015)**	**− 0.139 (0.019)**	**− 0.135 (0.024)**	**-0.140 (0.021)**
*Potentially inappropriate medicines*				
Beers	**− **0.054 (0.346)	**− **0.014 (0.793)	**− **0.017 (0.758)	**− **0.017 (0.753)
PRISCUS	**− **0.035 (0.536)	**− **0.031 (0.573)	**− **0.034 (0.541)	**− **0.030 (0.593)
FORTA-D	**− **0.040 (0.488)	**− **0.034 (0.563)	**− **0.035 (0.561)	**− **0.057 (0.347)

Values are shown as standardised regression coefficient B and p-value. Bold print indicates a *p*-value < 0.05. * = inappropriate rating in at least 1 of the 9 criteria, CCS = Charlson Comorbidity Score. Model 1 is unadjusted. Model 2 is adjusted by age, sex, and respective value at admission. Model 3 is adjusted by age, sex, CCS, and respective value at admission. Model 4 is adjusted by age, sex, CCS, number of drugs at admission, and respective value at admission

**Fig. 2:**
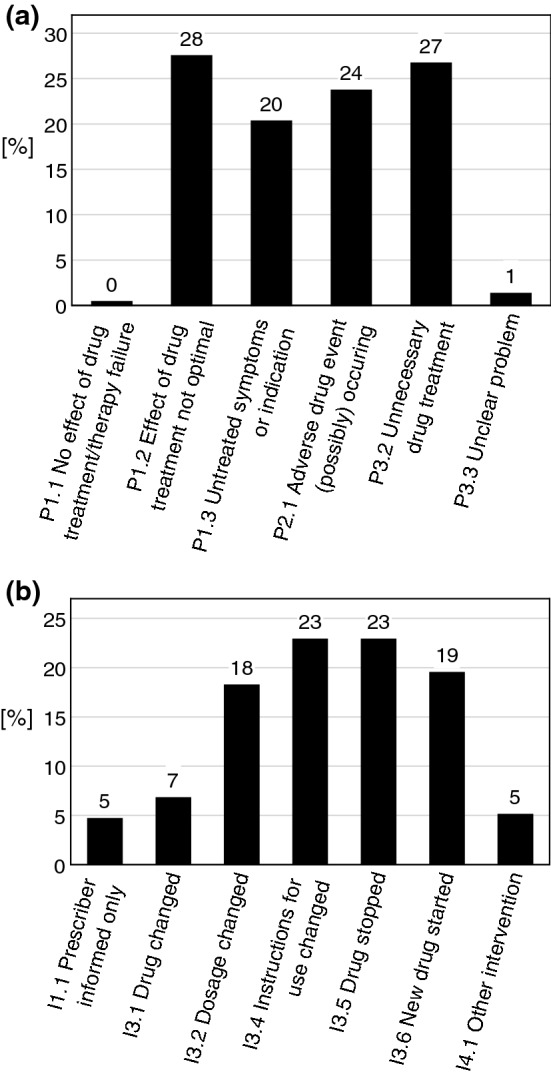
**a** Drug-related problems (DRP) initiating Pharmaceutical Interventions. Problems classified according to PCNE V8.01 [26]. *P* stands for Problem identified. P1.1–P1.3 concern the treatment effectiveness, P2.1 the treatment safety and P3.2–3.3 are other problems. **b** Pharmaceutical Interventions. Interventions classified according to PCNE V8.01 [26]. I stands for Intervention, the numbers denote the category, intervention is given in full

In total 236 DRP were identified and the resulting pharmaceutical interventions involved 104 patients (0–9 per patient). The identified DRP concerned the ‘treatment effectiveness’ (48%, *n* = 114), the ‘treatment safety’ (24%, *n* = 56) and ‘other problems’ (28%, *n* = 66) like unnecessary drug treatment (see Fig. 2a for more detail). Other problems were mainly ‘unnecessary drug treatment’ (27%, *n* = 63) and a few ‘unclear problems’ (1%, *n* = 3).

The most common causes of DRP were ‘no drug treatment despite existing indication’ (20%, *n* = 46), ‘inappropriate combination of drugs or drugs and herbal medication’ (13%, *n* = 31), drugs with ‘no indication’ (11%, *n* = 25) and ‘too frequent dosage regimen’ (11%, *n* = 26). The proposals for interventions of the clinical pharmacist ranged from ‘discontinuation of drugs’ (23%, *n* = 54) and the ‘change of instructions for use’ (23%, *n* = 54) to the ‘start of (new) drugs’ (19%, *n* = 46), the ‘change of dosage’ (18%, *n* = 43) and other interventions (totalled 17%, *n* = 39) (Fig. 2b).

Physicians accepted 88% of pharmaceutical interventions and fully or partially implemented 86%. Thus, 85% of DRP were totally or partially solved. For 4% it was either not possible or necessary to solve the DRP, for 1% it was unclear, whether the DRP was solved and for 1% the patient refused a change in their medication. For further 9% of DRP, the physician accepted the pharmaceutical intervention but did not implement it (2%) or rejected the intervention proposal of the pharmacist (7%).

**Fig. 3:**
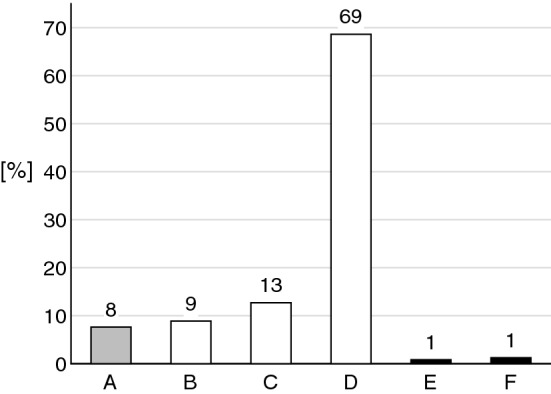
Proportion of DRP (drug-related problems) according to NCCMERP categories [31] as explained in Table 1. White bars indicate actual medication errors without patient harm (categories B, C, and D). Category A means no error occurred, categories E & F resulted in temporary harm and required an intervention (E) or hospitalization (F)

The majority of DRP, 90% (*n* = 213, possible discrepancies to 100% are due to rounding differences), were categorized as actual medication errors that did not result in patient harm (see Fig. 3). Of these, 9% (*n* = 21) of errors did not reach the patient, 13% (*n* = 30) reached the patient but did not cause patient harm and 69% (*n* = 162) reached the patient and needed monitoring or interventions like a change of drug treatment to make sure that the patient was not harmed. A further 8% (*n* = 18) were categorized as circumstances that have the capacity to cause error. The remaining 2% (*n* = 5) of DRP were categorized as errors that did cause temporary patient harm. Examples of these were pain without prescribed/administered analgesics, a sleep-inducing neuroleptic administered in the morning and bleeding at admission for a patient who took an inappropriate combination of drugs that can cause or worsen bleedings.

## Discussion

### Statement of key findings

This study demonstrates adding a pharmacist to the multi-professional team on a geriatric ward significantly increases the medication quality and safety, especially for correct dosage as well as correct and practical directions of medications. Pharmaceutical interventions are well accepted and implemented by physicians on the geriatric ward.

### Strengths and weaknesses

This study has several strengths and weaknesses. Due to the hospital set-up with one geriatric ward and one medical team a randomised study design was not possible. Hence, the before-after approach with a historical control period was the best possible alternative. Even without randomization, the two groups were comparable in size, sex distribution, morbidity, independence in ADL and MAI summated patient score. Mean age and the number of drugs at admission differed between the two groups. For both factors the intervention group was worse, *i.e.* the patients were older and took more drugs than the patients in the control group. One reason might be that the geriatric ward was well established during the intervention group but had only just started at the start of the control group.

Second, the MAI rating is a subjective rating. Since the same investigator did all the ratings, a potential bias would have been consistent over all groups and phases. To account for the same investigator providing the pharmaceutical care on the ward as well as the assessment of the MAI, there was a considerable time lag between providing the individual pharmaceutical care and the assessment of the MAI. Additionally, another investigator validated the ratings. Third, follow-up was not possible due to the study design and data protection constraints. Finally, the NCCMERP evaluation could be perceived as subjective and overestimating the harm experienced by patients. To counteract this, we chose to classify all ambiguous situations better than worse and all DRP classified with harm for the patient had that harm clearly documented in the medical notes. Two investigators assessed all DRP with a NCCMERP classification indicating harm for the patient. Although MAI, PCNE and NCCMERP can be perceived as subjective, they plus using several PIM lists, offer good opportunities to evaluate medication quality as objectively as possible and are well-established methods in similar studies. One strength of this study was the integration of the ward pharmacist before the intervention. That way, the pharmacist became an integral part of the multi-professional team and the other ward staff were used to the pharmacist and her expertise. Furthermore, the improvement in MAI was adjusted to several confounding factors and not only analysed within the two groups.

### Interpretation

The findings regarding the MAI are in line with several European studies that evaluated the changes of MAI with pharmaceutical interventions [18]. The significant improvements in the MAI criteria *Dosage, Correct Directions,* and *Practical Directions* reflect the expertise pharmacists add to a multi-professional team as these criteria refer to core knowledge of pharmacists. Other criteria within the scope of pharmacists’ expertise like *Drug-Drug Interactions* and *Duplications* already had a very low proportion of inappropriate ratings at admission, so an intervention effect is not visible. A contributing factor to some criteria showing significant improvement in the intervention group but not the summated patient score could be the different weight application to summated scores. Criteria like *Indication* and *Effectiveness* are weighted higher (each 3 points) than criteria like *Dosage,* or *Correct Directions* (each 2 points) and *Practical Directions* (1 point). This could be why the intervention did not show an effect on the summated patient score but on the overall proportion of inappropriate ratings. Another recent study in Germany reported even better improvements in MAI scores, but had significantly higher baseline MAI scores and weighted the criterion *Indication* lower than in the original instructions [17]. Also, the baseline MAI was different in the control and the intervention group, and the MAI improvement was not adjusted to other variables [19]. Additionally, the study used an undisclosed method of calculating the MAI, so it was not possible to compare their results to ours.

The good results for MAI and PIM reduction in the control group demonstrate an already excellent medical care by the multi-professional team on the geriatric ward, even without a pharmacist on ward. The setting of focused care for geriatric patients and longer hospital stays than usual show positive effects in itself. The physicians caring for patients in the present study were trained geriatricians or registrars in training to be geriatricians with a focus on geriatric pharmacotherapy. Therefore, the additional value of a pharmacist in other medical disciplines not trained in polypharmacy should be even higher.

The proportion of PIM was reduced from admission to discharge in both groups, control and intervention. No additional effect for the intervention could be demonstrated. This confirms the results of other studies with geriatric patients [28].

The DRPs found by the pharmacist during the study did originate in medication errors on the ward as well as medication errors from the primary care setting or on wards that treated the patient before the geriatric ward. The most common drugs missing despite existing indication were alendronic acid and cholecalciferol. Those medication errors became apparent once the patient was transferred on the geriatric ward. Similarly, the most common drug prescribed without indication was pantoprazole, normally initiated before the patient was transferred to the geriatric ward.

The high acceptance rate of pharmaceutical interventions (88%) demonstrates the clinical significance of DRP and the good inter-professional collaboration on the geriatric ward. The direct contact between the pharmacist and the multi-professional team and a well-established collaboration before the study contributed to the success and outcome of this study. This finding is confirmed by another study evaluating the satisfaction of physicians with a similar service for geriatric inpatients in Belgium [29]. The physicians’ satisfaction with the clinical pharmacy service was very high and the service was perceived as clinically relevant as well as time-saving [29]. Other studies already demonstrated that the acceptance of pharmaceutical interventions is higher the more integrated the pharmacist is within the ward team [30].

The classification of DRPs according to NCCMERP medication error categories demonstrates that pharmaceutical interventions were mainly preventive as most errors did not lead to patient harm. Medication errors categorised as errors that required initial or prolonged hospitalisation (NCCMERP-F) were all errors that originated in the primary care setting and caused the initial hospitalisation, as the errors caused or worsened mainly acute bleedings.

### Further research

Further research should be conducted regarding the sustainability of the pharmacist’s intervention after discharge and regarding similar pharmaceutical interventions for geriatric patients on non-geriatric wards with physicians who might even benefit more from the additional expertise pharmacists contribute.

## Conclusion

Although no effect on overall MAI per patient could be shown in our study, pharmaceutical care on ward improved the MAI criteria for *Dosage, Correct* and *Practical Directions* as well as the proportion of overall inappropriate MAI ratings (at least 1 of 9 criteria inappropriate) significantly. Combined efforts are necessary to improve the medication quality of elderly patients. The present approach has the potential to minimize risks and improve medication appropriateness in geriatric inpatients.

## Abbreviations

ADL: Activities of daily living
ADR: Adverse drug reactions
DRP: Drug-related problems
CCS: Charlson Comorbidity Score
MAI: Medication appropriateness index
NCCMERP: National Council for Medication Error Reporting and Prevention
PCNE: Pharmaceutical care network Europe
PIM: Potentially inappropriate medications
